# EXPERIENCES OF TECHNOLOGY USE BY OLDER ADULTS DURING COVID-19

**DOI:** 10.1093/geroni/igad104.2865

**Published:** 2023-12-21

**Authors:** Tina Kilaberia, Yuanyuan Hu, Edward Ratner, Janice Bell

**Affiliations:** New York University, New York City, New York, United States; New York University, New York City, New York, United States; Minneapolis VA Health Care System, Minneapolis, Minnesota, United States; University of California, Davis, Davis, California, United States

## Abstract

Computer and communications technology use expanded during the COVID-19 pandemic, but little is known about how this affected older people. Using the Unified Theory of Acceptance and Use of Technology questionnaire and qualitative interviews, we examined 18 older adults’ technology use during COVID-19 across two subgroups of nine older adults each: those scoring above (“high”) and those scoring below (“low”) the median (4.09). Overall, older adults scored fairly high (mean 4.0; SD=0.5; range 3.1-4.8 across 16 items on a scale of 1 to 5). In each group of nine older adults, four (44%) described technology as being helpful during the pandemic. Among high scoring older adults, 100% utilized technology to order either groceries or household items and six (67%) used either telehealth or online health information system (i.e., MyChart) to communicate with health services providers, compared to three (33%) and four (44%) within the low scoring group. No one in the high scoring group described a degree of cognitive difficulty whereas four 44% in the low scoring group did. All but one (94%) older adults described needing some help with technology from family members, friends, and/or paid technology services. Two themes complement these findings by reflecting strengths (“lots of ways to do everything”) and challenges (“I just get through it”) of using technology. Greater technology mastery may positively influence the ability of older adults to meet their needs, but cognitive challenges may prevent full engagement with technology.

